# Supplementary material for: Identification of photoperiod-induced specific miRNAs in the adrenal glands of Sunite sheep (Ovis aries)
Source: Front Vet Sci. 2022 Jul 22;9:888207. doi: 10.3389/fvets.2022.888207 (PMC9354845; doi:10.3389/fvets.2022.888207)
Supplement: Supplementary file 1 [file Data_Sheet_1.ZIP › Supplementary file/miRDeep2 file.zip!/miRDeep2 file/Know_Structure/LP42A3/oar-mir-181a-1.pdf]

miRBase precursor : oar-mir-181a-1
Total read count : 20376
oar-miR-181a read count : 20291
remaining reads : 85

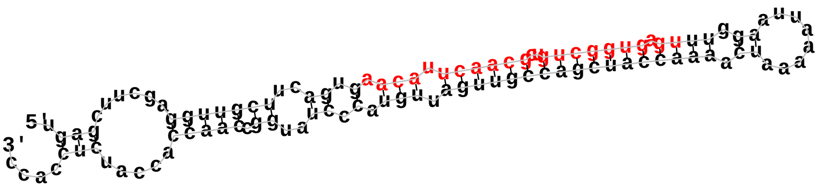

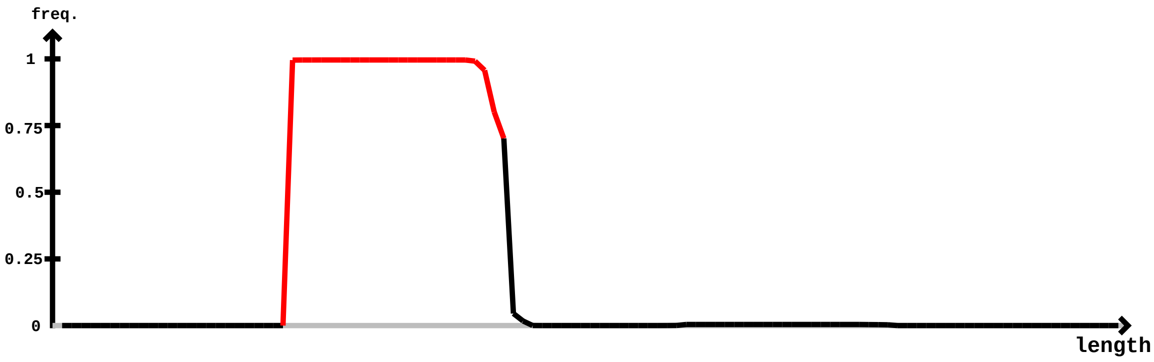


```
                        oar-miR-181a
5'- ugagcuucgagguuguucagugaacauucaacgcugucggugaguuuggaauuaaaaaucaaaaccaucgaccguugauuguacccuauggccaaccaccaucuccacc   -3'   exp
    .(((......(((((((..((.(.(((.((((((..(((((((.((((.((........)).))))))))))))))))).))).).))..)).))))).....)))....    reads     mm     sample
    ......................aacauucaacgcugucgg.....................................................................    1         0      seq
    ......................aacauucaacgcugucggu....................................................................    89        0      seq
    ......................aacauucaacgcugucggug...................................................................    698       0      seq
    ......................aacauucaacgcugucgguga..................................................................    3183      0      seq
    ......................aacauucaacgcugucggugag.................................................................    2022      0      seq
    ......................aacauucaacgcugucggugagu................................................................    13369     0      seq
    ......................aacauucaacgcugucggugaguu...............................................................    569       0      seq
    ......................aacauucaacgcugucggugaguuu..............................................................    346       0      seq
    ......................aacauucaacgcugucggugaguuug.............................................................    4         0      seq
    ......................aacauucaacgcugucggugaguuuggaauu........................................................    1         0      seq
    .......................acauucaacgcugucggugag.................................................................    1         0      seq
    .......................acauucaacgcugucggugagu................................................................    8         0      seq
    .........................auucaacgcugucggugagu................................................................    1         0      seq
    .............................................................aaaccaucgaccguugauugu...........................    2         0      seq
    .............................................................aaaccaucgaccguugauugua..........................    1         0      seq
    ..............................................................aaccaucgaccguugauugua..........................    1         0      seq
    ..............................................................aaccaucgaccguugauuguac.........................    2         0      seq
    ..............................................................aaccaucgaccguugauuguacc........................    3         0      seq
    ...............................................................accaucgaccguugauugu...........................    6         0      seq
    ...............................................................accaucgaccguugauugua..........................    4         0      seq
    ...............................................................accaucgaccguugauuguac.........................    10        0      seq
    ...............................................................accaucgaccguugauuguacc........................    55        0      seq
```
